# Beyond DNA repair and chromosome instability—Fanconi anaemia as a cellular senescence-associated syndrome

**DOI:** 10.1038/s41418-021-00764-5

**Published:** 2021-03-15

**Authors:** Anne Helbling-Leclerc, Cécile Garcin, Filippo Rosselli

**Affiliations:** 1 UMR9019-CNRS, Gustave Roussy, Villejuif, Cedex France; 2 Université Paris-Saclay, Orsay, France; 3 Equipe labellisée “La Ligue Contre le Cancer”, Villejuif, France

**Keywords:** Cancer genetics, Disease genetics

## Abstract

Fanconi anaemia (FA) is the most frequent inherited bone marrow failure syndrome, due to mutations in genes encoding proteins involved in replication fork protection, DNA interstrand crosslink repair and replication rescue through inducing double-strand break repair and homologous recombination. Clinically, FA is characterised by aplastic anaemia, congenital defects and cancer predisposition. In in vitro studies, FA cells presented hallmarks defining senescent cells, including p53-p21 axis activation, altered telomere length, mitochondrial dysfunction, chromatin alterations, and a pro-inflammatory status. Senescence is a programme leading to proliferation arrest that is involved in different physiological contexts, such as embryogenesis, tissue remodelling and repair and guarantees tumour suppression activity. However, senescence can become a driving force for developmental abnormalities, aging and cancer. Herein, we summarise the current knowledge in the field to highlight the mutual relationships between FA and senescence that lead us to consider FA not only as a DNA repair and chromosome fragility syndrome but also as a “senescence syndrome”.

## Facts

- Unrestricted activation of the DDR signaling leads to a constitutive activation of the growth inhibitory p53-p21 axis in Fanconi anemia (FA) cells.
- Several hallmarks of senescence, including telomere’s abnormalities, ROS overproduction, altered nuclear structure, overproduction of several pro-inflammatory lymphokines, cytokines and growth factors are classically observed in FA cells.
- FANCA, FANCD2 and BRCA1 are actively degraded to allow senescence progression induced by oncogene activation.

## Open questions

- Is the overactivation of the p53-p21 axis in FA that is, in fine, responsible for the major clinical and cellular stigmas of the syndrome?
- What is the role of the pro-senescent phenotype of the FA cells in the bone marrow failure of the patients?
- How FA cells surround their growth inhibitory status to become tumoral?
- Is the pro-senescent phenotype of the FA syndrome a target for therapeutic approaches?

## Introduction

Cellular senescence is a genetic process allowing proliferation arrest with physiological roles in embryogenesis, the maintenance and regeneration of tissues or the defence mechanism against tumours. By contrast, its deregulation has been primarily implicated in pathological processes, such as accelerated aging, aged-associated disease and tumorigenesis [1]. DNA damage and DNA damage signalling have been recognised as key and general triggers of senescence initiation and maintenance [2, 3].

Fanconi anaemia (FA), the most frequent inherited bone marrow failure syndrome (iBMFS), is characterised by congenital defects and leukaemia predisposition [4]. FA occurs because of mutations in genes encoding the FANC/BRCA pathway proteins involved in DNA interstrand crosslink (ICL) repair and replication rescue through managing one-ended or double-ended double-strand breaks (DSB) repair via Break-Induced Replication (BIR) or homologous recombination (HR) [5, 6]. FA is genetically and clinically heterogeneous [7]. Thus, we currently have a largely imperfect understanding of the link between the DNA damage response and DNA repair alterations of FA cells and their cellular and clinical outcomes. Potential alterations in the cellular senescence programme could represent a missing link between genetics and pathophysiology in FA.

Herein, we summarise the current knowledge on both senescence and FA to highlight the mutual relationships between the two fields that led us, *in fine*, to consider FA not only as a DNA repair and genome instability syndrome but also as a “senescence syndrome”.

## Cellular senescence

**Fig. 1:**
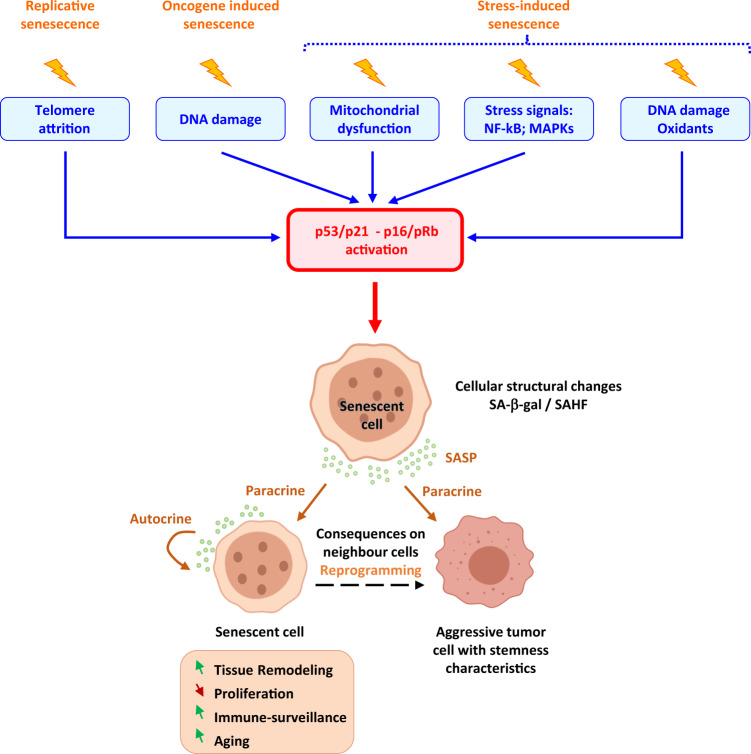
Major molecular and cellular causes of senescence implantation and consequences of SASP secretion on neighbour cell reprogramming. Many factors lead to senescence implantation, such as telomere replication, oncogene activation, damaging stimuli and mitochondrial dysfunction. These factors all lead to DNA damage persistence, ROS (reactive oxygen species) increase and stress pathway activation, which drive permanent growth arrest and cellular structural changes. When senescence is engaged, these cells express SA-β-gal (senescence-associated β-galactosidase) and SAHF (senescence-associated heterochromatin foci). They actively communicate with their microenvironment through the SASP (senescence associated-secretory phenotype). Depending on its composition, secreted factors can either drive both the autocrine and paracrine induction of senescence, immunoclearance, and tissue remodelling or enhance the aggressiveness of neighbouring tumour cells.

A senescent cell presents a multifaceted phenotype showing permanent growth arrest, resistance to apoptosis, enhanced secretion of several lymphokines, cytokines and growth factors (senescence-associated secretory phenotype, SASP), macromolecular damage and altered metabolism. Cell senescence has two opposing faces: one physiological acting during embryogenesis, tissue remodelling and repair, normal aging and tumour suppression, and the other pathological, acting as a driving force for degenerative diseases and cancer [1, 8, 9]. Given this ambivalence, aging and senescence are not synonymous. The first defines a process at the level of the organism and the second defines molecular and biological events at the tissue or cellular level [10]. Three major causes of cellular senescence have been described: replicative senescence, oncogene-induced senescence (OIS) and stress-induced senescence (Fig. 1). Whatever the initial input, the activation of the p53/p21 and the p16/Rb pathways is a key event in the implementation of the senescence program.

### Origin of senescence

**Fig. 2:**
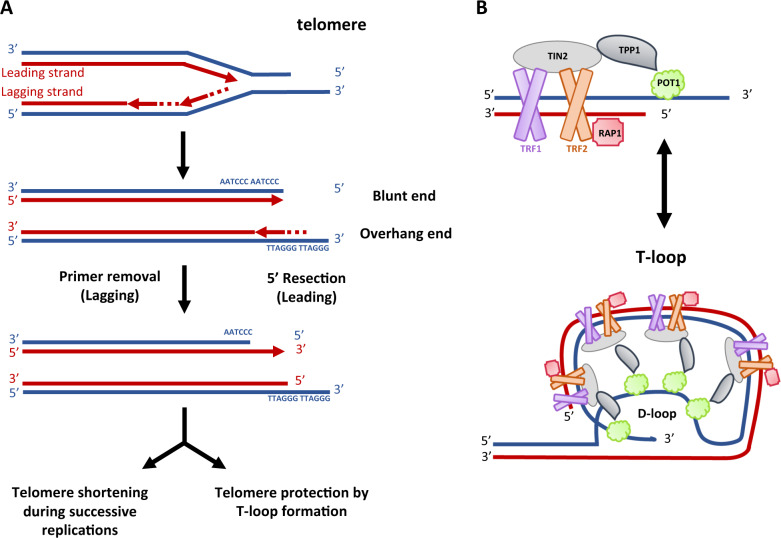
Structure of the telomere. **A** Replication of telomeres which shorten at each cell division and terminates with a 3-ssDNA-end G-rich overhang extremity. **B** Telomeres, located at the chromosome termini, are capped by the shelterin complex comprising six proteins. TRF1 and TRF2, which form homodimers, interact with the telomeric dsDNA form whereas POT1 is associated with telomeric ssDNA at the 3′ overhang. TIN2 acts as a bridge between these two homodimers. The telomere likely exists in a dynamic equilibrium as a linear structure with a free 3′ overhang (to the top) and the T-loop structure (below).
- **Oncogene-induced senescence**
  The unrestrained activation of an oncogene induces cellular senescence as a consequence of the DNA hyper-replication-associated DNA damage which leads to a permanent DDR activation: a phenomenon called OIS [2, 3]. Even if activating mutations in *RAS* or *BRAF* are commonly observed in many human cancers, their sole activation is not sufficient to drive transformation and requires additional hits. In their absence, unscheduled oncogene activation leads to growth arrest and cellular senescence, demonstrating that senescence *in fine* represents an efficient antitumor mechanism. OIS is accompanied by the expression of tumour suppressors, such as p53, p16^INK4a^ and pRb, whose loss-of-function leads to the abrogation or bypass of the senescence programme [17, 18].
- **Stress-induced senescence**
  Finally, several other stressful stimuli induce premature senescence again *via* the activation and permanent maintenance of DDR signalling. They constitute a heterogeneous group of events: alterations in DNA methylation and/or histone landscape [19], cell exposure to pro-inflammatory cytokines or the SASP produced by neighbouring cells, oxidative stress or reactive aldehyde due to endogenous cellular metabolism [20], mitochondrial and metabolic dysfunctions [21] (Fig. 1). Offering an interesting exemple of the intricate links between metabolic pathways, mitochondria activity, DNA damage and redox homeostasis in cellular senescence, a deficiency in the Alcohol Dehydrogenase 5 (ADH5), which is critical for formaldehyde clearance, associates alterations in mitochondrial dynamics and mitophagy as well as increased ROS and DNA damage (induced by the excess of oxidised aldehydes that react with DNA) which converge to cellular senescence [22, 23].

- **Replicative senescence**
  Replicative senescence is due to replication-associated telomere shortening. The telomere, the extremity of the eukaryotic chromosome, comprises repetitions over a length of 5–20 Kb of the DNA sequence TTAGGG. They cannot be fully replicated by DNA polymerases because of the “end replication” problem [11]: chromosomes shorten at each cell division and terminate with a 3′-ssDNA-end G-rich overhang extremity, which resembles a resected one-ended DSB. The shelterin complex, which includes TRF1, TRF2, POT1, RAP1, TIN2 and TPP1 proteins, coats and caps telomeric DNA mediating the folding back of the 3′-ssDNA-end G-rich overhang inside the dsDNA region that precedes it, leading to a T-loop structure that “closes” the chromosome extremity (Fig. 2) [12]. Shelterin ensures telomere protection against extensive resection impeding the activation of a pernicious DNA damage response (DDR) that would manage a telomere erroneously as a one-ended DSB [13]. Moreover, TRF2 inhibits ATM kinase-stimulated CtIP/MRN resection and TPP1/POT1 inhibits ATR kinase-stimulated EXO1/BLM resection. Inactivating mutations affecting one protein of the shelterin complex leads to the constitutive activation of ATM- or ATR-dependent DNA damage signalling [13]. Thus, even if telomeres shorten at each cell cycle, shelterin complex maintains their genetic integrity and functionality during around 40–60 cell divisions, the so-called “Hayflick limit” [14]. Furthermore, the short and shelterin-unprotected telomeres are managed by DNA repair pathways, leading to telomere end-associations and consequent post-mitotic DSB accumulation, which, by switching-on constitutionally the DDR, imposes a permanent cell cycle arrest. Indeed, telomeres that are critically eroded present telomere dysfunction-induced foci (TIFs) that reflect the accumulation of 53PB1 on uncapped telomeres. Telomere shortening is a major determinant of lifespan and longevity [15]. In cancer cells, which maintain “indefinitely” a high proliferative activity and escape to senescence, the telomere’s length and functionality are maintained by two alternative mechanisms: re-activation of the telomerase, a ribonucleoprotein complex with reverse transcriptase function associated with an RNA template and several other proteins (which are turned off in human somatic cells) or a HR mechanism known as alternative lengthening of telomeres, observed in ~10–15% of human cancers [15, 16].

### Senescence: from physiology to pathology

The physiological requirements of senescence have been proposed for many biological processes, such as tissue remodelling during embryogenesis, tissue repair and immune-surveillance [1]. As revealed by the presence of SA-β-gal (senescence-associated β-galactosidase) positive cells, senescence occurs during embryonic development to sculpt the organism. During embryogenesis, senescence is p21 dependent but p53, p16 and DNA damage independent and is regulated by the TGF-β/SMAD and the PI3K/FOXO pathways. Senescent cells are cleared by macrophages, allowing tissue remodelling [24, 25]. Tissue repair is a process comprising four phases: haemostasis, inflammation, proliferation and remodelling. Initially, senescence is detected in fibroblast and epithelial cells early in response to an injury. The senescent cells accelerate wound closure by inducing myofibroblast differentiation through the secretion of PDGF-AA [26]. However, an excess of senescent cells after an injury may cause organ failure and/or permanent illness. Extracellular signalling mediated by senescent cells through the SASP can induce stem cell activation, which promotes tissue regeneration and cellular plasticity [27]. Finally, premalignant senescent cells are removed from healthy tissue by an efficient immune-surveillance system that entails both the innate and adaptive immune response through the “senescence surveillance” pathway [28]. Accordingly, senescence activation in a RAS-induced carcinoma mouse model resulted in the rapid regression of the existing tumour. Senescence is a cellular defence mechanism that can rapidly stop the abnormal proliferation of cells with mutated oncogenes or cells that acquire irreversible damage.

By contrast, the accumulation of senescent cells associated with chronic inflammation causes disease, aging and cancer. In advanced age, senescent cells accumulate in the organism because of several factors, including declining immune function and senescent cells clearance, DNA damage accumulation and the inability to stabilise p53 to the levels required to cause apoptosis [29–32]. The use of mice in which positive p16 cells are selectively targeted for elimination has unequivocally demonstrated that persistence/accumulation of senescent cells impacts negatively individual well-being and lifespan [33]. Moreover, retention of unwanted senescent cells lead to the accumulation of SASP components, particularly TGF-β, TNF-α, IL-6 and IL-8 cytokines, which induce or support transformation, epithelial–mesenchymal transition and invasiveness [34–36]. Thus, senescence can have two opposite outcomes in cancer, acting against tumours and promoting their progression [37, 38]. In progeroid and/or neugenerative diseases [39], premature/excessive senescence has been associated also to replication stress or altered reponse to DNA damage. Indeed, it has been reported that progerin accumulation in the Hutchinson-Gilford Progeria Syndrome affects the localisation of PCNA, the processivity factor of the DNA polymerase δ, affecting DNA replication and leading to replication stress and the subsequent p53 activation [40]. Similarly, in the neurodegenerative diseases and UV-sensitive disease Cockayne syndrome, mutations in the nucleotide excision repair component CSB/ERCC6 leads to the upregulation of the p53-p21 axis, cause and hallmark of senescence (see below) [41, 42]. In addition, there is extensive evidence indicating an involvement of senescent cells accumulation in aggravate the neurodegeneration in Parkinson’s, Alzheimer’s diseases, and Down syndrome [43].

### Senescence hallmarks

Despite their heterogeneity, several hallmarks define senescent cells [8, 38].

- **ATM and ATR activation**
  The major common biomarker that defines senescent cells is the presence of persistently activated DDR due to the accumulation of irreparable DNA lesions. For exemple, a linear uncapped or eroded telomere looks like a DSB and activates ATM kinase [44]. Its “repair” results in end-to-end chromosome fusions or fusion with a DSB extremity elsewhere in the genome, resulting in a dicentric chromosome that will lead to a new DSB after its breakage during mitosis. Thus, cells with eroded telomeres maintain the ATM-dependent cell cycle checkpoint that arrests proliferation in the “on” state [45]. Oncogene unrestrained activation is followed by a hyper-proliferative phase associated with an increased number of active replicons and fork instability, leading to robust S-phase-specific DDR engagement through both ATM and ATR activation [2, 3]. Because the oncogenic signals cannot be silenced, ATM/ATR signalling is permanently maintained, inducing senescence entry. The enforcement of DDR, which is dependent on DNA replication, is both causative and necessary to initiate and maintain OIS, opposing cell transformation [2, 3]. Finally, most cellular stresses, including exogenously induced DNA damage, endogenous oxidative stress, mitochondrial impairment, changes in the chromatin landscape and exposure to the SASP, affect the DNA chemistry or structure. Therefore, while a mis-repaired DNA lesion can drive transformation to increase the mutational landscape of a cell, persistent DNA lesions lead to senescence by hampering replication. In the absence of effective DDR, senescence is bypassed, allowing abnormal cell proliferation and transformation. Effectively, key DDR players are progressively lost during cancer evolution [46].
  In summary, DDR activation and senescence represent initial barriers to oncogene-induced proliferation and, to progress, cancer needs to bypass these barriers. Thus, DDR signalling appears to be an important marker and contributor to the cell’s decision to undergo senescent.
- **Activation of the p53-p21 and p16-pRb axes**
  Driven by ATM/ATR, DDR signalling leads to cell cycle arrest by activating the p53-p21 and p16-pRb axes, which inhibit factors associated with the G1-S transition. Both axes play critical and pleiotropic roles in growth inhibition outcomes: arresting the cell cycle temporarily and permitting DNA damage repair or permanently stopping the cell proliferation of highly damaged cells by inducing senescence or cell death [47].
- **Cellular structure alterations**
  Senescent cells become enlarged due to mTOR pathway activation [48] and acquire an irregular shape caused by the overexpression of vimentin filaments that alters the cytoskeleton.
  The plasma membrane composition is also modified. Recent wide screening revealed that no less than one hundred plasma membrane proteins could represent potential senescence markers and their presence correlates with survival increase in different tumours [49].
  The upregulation of lysosomal proteins was detected in senescent cells due to old lysosome accumulation, increased lysosomal biogenesis and overexpression of specific proteins, such as SA-β-gal, which is the product of one of the multiple transcripts of the *GLB1* gene encoding lysosomal β-D-galactosidase [50]. The evaluation of SA-β-gal activity is a commonly used readout for senescent cells in culture and mammalian tissues, despite the variability of its expression.
  Generally, the senescent cell’s nucleus is enlarged, exhibiting senescence-associated heterochromatin foci (SAHF) observed as punctate DAPI foci visible by microscopy. In addition to being enriched in heterochromatin markers such as H3K9me3, HP1 and histone macroH2A, an increased level of HMGA proteins is required for SAHF formation [51]. Heterochromatin remodelling has been proposed to be exploited by the cell to prevent the transcription of E2F target genes, which are associated with S-phase entry and cell proliferation [52]. In senescent cells, the nuclear envelope structure is altered because of p53- and p16-dependent downregulation of lamin B1 [53]. This event affects the spatial reorganisation of chromatin and gene expression [51, 54, 55]. The loss of integrity of the nuclear envelope causes a release of chromatin pieces from the nucleus to the cytoplasm. These cytoplasmic chromatin fragments (CCFs) are processed using an autophagic/lysosomal pathway [56].
  Senescent cells also show mitochondrial alterations caused by a mitochondria number increase due to mitophagy reduction. This dysfunction is accompanied by a significant mitochondrial potential membrane decrease, ROS (reactive oxygen species) increase and oxidative DNA damage [21, 57].
- **Genetic and epigenetic regulation**
  With aging, genes associated with the stress response are up-regulated, while genes involved in maintaining genome integrity, including DNA repair genes, are down-regulated [58]. Accordingly, the efficiency of DNA repair in aged cells is reduced, leading to gradual DNA damage accumulation and permanent DDR that activates the senescence programme.
  Histone and DNA methylation events are also associated with senescence. DNA methylation of constitutive heterochromatin is decreased by DMNT1 downregulation, but local hypermethylation of CpG islands is observed at the promoter-proximal regions of cell cycle genes associated with their repression [19]. For example, the repressive histone mark H4K20me3 is enriched on the pro-apoptotic gene *Bax* in response to senescence-associated oxidative stress [59].
  In senescent cells, some genomic regions acquire a more “open” structure, such as chromatin hosting the major retrotransposon classes Alu, SVA and L1, and, constitutive heterochromatin in centromeric and peri-centromeric regions [60]. These features have also been observed in cancer cells. Thus, premalignant senescent cells undergo changes in methylome that cause cancer progression when senescent cells can escape the proliferative barrier [19].
- **Secretory phenotype**
  Senescent cells secrete many factors, such as cytokines (IL-1α, IL-6, IL-13), chemokines (IL-8, CCL2), inflammatory molecules (TGF-β, IFN-γ), proteinases (MMP-14, MMP-7, MMP-3) and growth factors, which regulate several biological process [61]. The SASP is highly heterogeneous and dependent on the cell type and senescence origin. It is also the result of a transcriptional programme mediated by different factors. Persistent DDR activation is associated with pro-inflammatory transcription factor NF-κB activation or with p62-mediated autophagy reduction, which, in turn, inhibits GATA4, recently described as a senescence regulator. Interestingly, the ATR/p62/GATA4 axis is independent of p16^INK4a^ and p53. GATA4 stabilisation indirectly activates NF-κB to initiate and maintain the SASP [62]. The p38/MAPK axis is also involved in the NF-κB-dependent pro-inflammatory activity required for SASP secretion [63]. Thus, p38/MAPK axis upregulation induces the overexpression of matrix metalloprotease MMP7 and activates IL-8 and TNF-α oversecretion [64].
  Recently, it has been shown that the recognition of CCF by cGAS triggers the production of SASP factors via STING, thereby promoting paracrine senescence [65, 66].
  Proteases are required for senescence progression. It is the case of cathepsin-L1 (CTSL1) which degrades 53BP1, a key protein for DNA repair by non-homologous end-joining (NHEJ), and cleaves the tail of histone H3.3, facilitating the transcriptional silencing of cell cycle regulators, including some E2F target genes [67, 68].
  Depending on the physiological context, SASP factors can either reinforce senescence growth arrest in an autocrine manner or relay the senescence phenotype to surrounding cells in a paracrine manner (Fig. 1) [34]. The SASP also contributes to the surveillance and elimination of senescent cells by the immune system. Thus, paracrine senescence mediates the beneficial effects of senescent cells on tissue homeostasis. However, chronic expression of SASP proteins leads to a disease state or an aging phenotype [17, 30, 33]. The SASP, through TGF-β, TNF-α, IL-6 and IL-8, also induces transformation, the epithelial–mesenchymal transition and invasiveness [34]. Senescent cells can alter the tissue microenvironment affecting neighbouring cells through paracrine signalling, leading to angiogenesis stimulation by senescent fibroblasts, altering epithelial cell differentiation and promoting the growth and tumorigenesis of epithelial cells [69, 70].
- **Metabolic changes**
  Senescent cells remain metabolically active. Several catabolic pathways are activated to stop futile DNA repair activities. p53 represses PGC1A and PGC1B expression, leading to mitochondrial biosynthesis arrest and mitochondrial activity decrease, which drive the increase in the AMP:ATP and ADP:ATP ratios and AMPK (a central sensor of energy homeostasis) activation. Activated p53 also modifies glucose uptake and glycolysis, promoting the tricarboxylic acid cycle, oxidative phosphorylation and fatty acid oxidation [21, 71]. These metabolic changes converge to increased intracellular ROS levels, which are mitogenic signalling molecules that fuel oncogene-driven aberrant cell proliferation [72].

## Fanconi anaemia

### FA phenotype

Fanconi anaemia is a rare genetic disease affecting 1–4 newborns per million births and is the most frequent iBMFS. FA is also associated with several diverse features, all of which show incomplete penetrance, such as endocrine dysfunction, congenital abnormalities in several unrelated organs and cancer predisposition to acute myeloid leukaemia and solid tumours (particularly squamous cell carcinoma of the head and neck) [4, 73]. Around 3/4 of FA patients present at least one physical abnormality included in the VACTERL-H association (Vertebral, Anal, Cardiac, Trachea-esophageal fistula, Esophageal atresia, Renal upper Limb and Hydrocephalus) and PHENOS acronym (skin Pigmentation, small Head, small Eyes, Nervous system, Otology, Short stature). The most frequent traits associated with ~30% of the FA patients include short stature, radial ray defects, skin pigmentary changes, renal malformations, and microencephaly [7]. Long considered a DNA repair disease, FA has been recently presented as an “accelerated aging disease” due to the presence of clinical phenotypes such as osteoporosis, ventriculoperitoneal shunts, erythrophagocytosis and type II diabetes mellitus [74]. Nevertheless, the biochemical and molecular bases of the potentially accelerated organismal aging and their consequence in FA were not defined.

The major hallmarks of FA cells include chromosomal fragility and hypersensitivity to DNA ICL-inducing agents such as mitomycin C, diepoxybutane and cisplatin. The FA diagnosis is realised in vitro by the chromosome breakage assay in lymphocytes or fibroblasts exposed to ICL-inducing agents, which reveal high levels of typical chromosome aberrations, such as tri- and quadri-radials [4, 73].

### FANC proteins

**Fig. 3:**
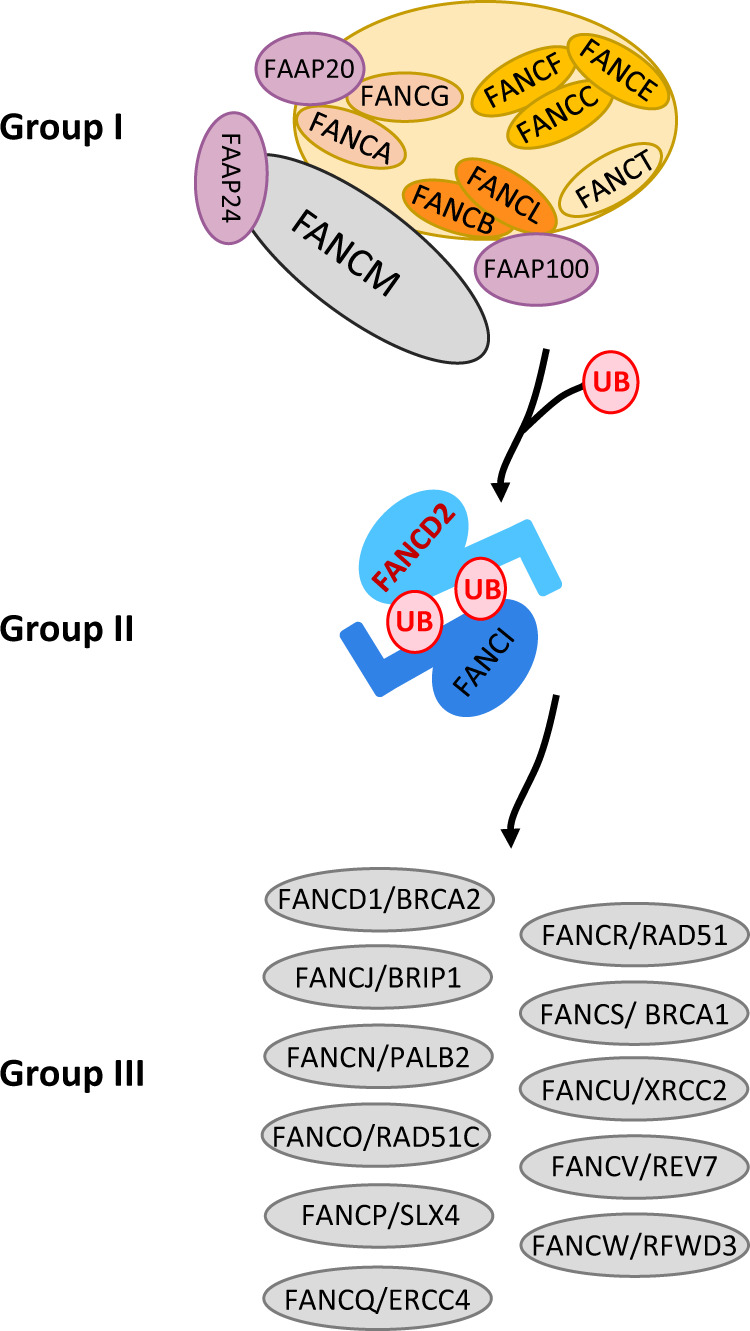
FANC/BRCA pathway. Schematic representation of FANC proteins in the three groups: proteins of group I associated with FAAP proteins constitute the FANC core complex, which allows monoubiquitylation of the FANCD2-FANCI complex (group II), which enables DNA incision, TLS (translesion synthesis), ICL elimination and replication rescue by homologous recombination, functions performed by group III FANC proteins.

**Fig. 4:**
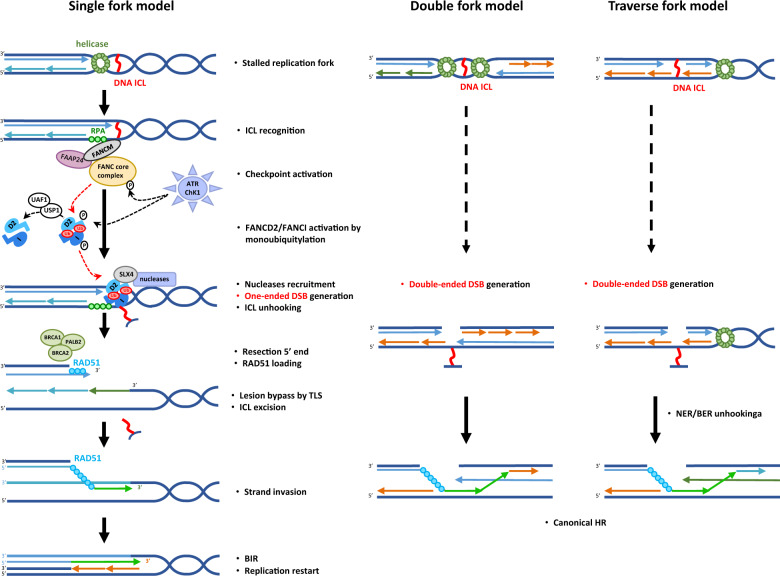
Repair of stalled forks by the FANC/BRCA pathway due to ICL. Different models are proposed in literature. In “single fork” or “double forks” models, replication forks are stalled at DNA ICL and recognised by FANCM-FAAPs (FAAP24). FANCM promotes the ATR-kinase dependent checkpoint response. The FANC core complex is activated and monoubiquitylates the FANCD2-FANCI complex. FANCI-FANCD2-ub complex and SLX4 are located in the chromatin and promote SLX4/nuclease activities in ICL unhooking. A double-strand break (one-ended or double-ended) is generated to allow subsequent resection and strand invasion by homologous recombination mediators in a process named break-induced replication (BIR) or canonical HR. In “traverse” model, FANCM after FANCD2 recruitment on ICL translocates in another side by MCM interaction to continue DNA synthesis. ICL repair is postreplicatif.

Currently, 22 mutated genes (*FANCA-FANCW* ) have been identified in FA patients. Proteins encoded by these genes compose FANC pathway which is biochemically and functionally organised into three groups (Fig. 3) [5, 75] and involved in several functions related to DNA metabolism, including ICL repair, replication fork protection and restart [76, 77]. Three models have been proposed to manage ICL repair (Fig. 4) [6]. The key points of the “single fork model” are arrest and collapse of one replication fork, induction of an one-ended DSB, ICL unhooking, TLS-dependent replication to complete replication of the strand with the unhooked ICL that will be successively “repaired” by NER/ BER-mediated elimination, and, finally, the HR-mediated (BIR) rescue of replication [78]. The converging “double forks model” is derived from in vitro reconstitution of the ICL repair steps [79] but it seems be a minor ICL repair mechanism in mammalian cells, estimated to 5–15% by Huang and collaborators [80, 81]. The most recent model proposed by these last authors, is based on DNA-combing technique, which allows the direct monitoring of DNA synthesis. The replisome seems able to reassemble on the other side of the ICL (“ICL traverse model”) to continue replication, an event largely dependent on the translocase activity of FANCM. ICL will be removed later by a BER/NER- and TLS-mediated mechanisms. ICL traverse has been observed in 50–60% of DNA fibers [80, 81]. Whatever the model, final ICL removal and DNA structural reconstitution are dependent on DSB formation (one-ended or double-ended) and repair via HR.

FANC pathway first group comprises eight FANC proteins (FANCA, B, C, E, F, G, and L) and forms the FANC core complex with FA-associated proteins (FAAPs). Assembled at the chromatin on FANCM, the multisubunits of the FANC core complex acts as a ubiquitin E3 ligase (activity driven by FANCL in association with FANCT). Combined with the ATR- and CHK1-mediated phosphorylation of FANCE, D2 and I, the FANC core complex ubiquitin-ligase activity monoubiquitylates FANCD2 and FANCI (the group II). Monoubiquitylated ID2 heterodimer re-localises on chromatin damaged sites, orchestrating the recruitment and function of group III proteins that allow DNA incision, TLS (translesion synthesis), ICL elimination and replication rescue by HR-mediated mechanisms (Figs. 3 and 4) [6].

Loss-of-function in the FANC pathway leads to several mitotic and post-mitotic abnormalities, including chromosome aberrations, anaphase bridges, lagging chromosomes and micronuclei [82]. Moreover, FANCD2 can interact directly with MCM proteins involved in replication [83] and promotes alternative end-joining DNA repair by recruiting POLθ [84]. FANCA plays a direct role in DSB repair, independent of HR, by catalysing single-strand annealing (SSA) and strand exchange [85]. These previous observations confirm the key role of FANC genes to prevent DNA breakage and rescue stalled replication forks.

Furthermore, a functional FANC pathway is important to protect specific regions of the genome called common fragile sites (CFSs) where large genes are located [86, 87], by managing conflict between transcription and replication because it protects cells from unscheduled accumulation of R-loops (DNA:RNA hybrid) [88]. In addition, FANCJ, with a helicase function is involved in maintenance of genome stability by recognition of specific DNA structure named G-quadruplexes (G4) which interfere with DNA replication, repair and mRNA transcription [89].

Subtle defects in immunity were observed in patients and recently reported in *Fanca*^*−/−*^ mice [90, 91].

### Is FA a cellular senescence-associated disease?

**Fig. 5:**
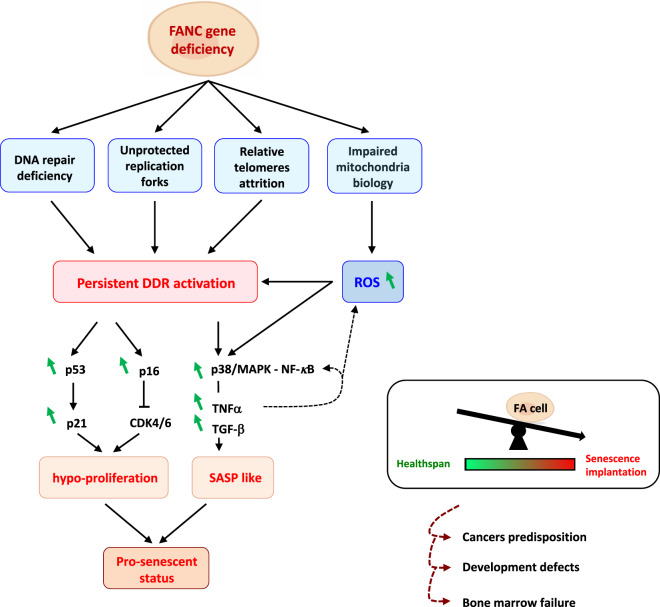
Fanconi Anaemia as a “senescence disease”. Fanconi anaemia cells are hypersensitive to multiple endogenous and exogenous stresses. The FANC pathway deficiency and impaired alternative roles of the FANC actors lead to persistent DDR activation and consequent stress pathway activation (p53/p21, p16, NF-κB, p38/MAPKs) and cell cycle arrest. The impaired DNA repair, accumulating defective mitochondria, elevated ROS levels and persistent inflammation factors (TNFα and TGF-β) contribute to exacerbate the stress pathway activation. This metabolic event leads to the pre-senescent status of FA cells, which could explain development defects, cancer predisposition and bone marrow failure observed in this disease (“dark side of senescence”).

A rapid survey of the characteristics of cells with FANC pathway deficiency allows the identification of several key hallmarks of senescence, including cellular hypo-proliferation, a short lifespan of fibroblasts in vitro, ATM, p53, p21 and p16 signal activation, and expression of SAHF and SA-β-gal (Fig. 5) [92, 93].

### Indirect evidence

- **Unrestricted activation of DDR signalling**
  The first argument that links FA to altered senescence is the persistent DDR activation observed in the patient’s cells. The main direct consequence of FANC pathway loss-of-function is the accumulation of DNA breaks at stalled/delayed replication forks. Such breaks activate DDR dependent on ATM or ATR, triggering the formation of several chromatin-associated DNA repair foci, assembling γH2AX, 53BP1, RIF1 and RAP80, and the activation of the growth inhibitory pathway [92, 94]. The subtly but well-described constitutive activation of the ATM-p53-p21 axis and ATR-CHK1 pathway is involved in cell cycle delay and the activation of both senescent and apoptotic programmes in FA. The unscheduled and unrestrained activation of the p53-p21 axis, characterised by several iBMFS, is responsible for the FA-associated haematological phenotype. p53 knockdown (which per se leads to increased genomic instability and DNA damage [95]) rescues the hematopoietic defects in *Fancd2*^*−/−*^ or *Fanca*^*−/−*^ murine bone marrow [96, 97], suggesting that BMF in FA is due more to aberrant DNA damage signalling conveying growth-inhibitory directives than to DNA damage per se.
- **Relative attrition telomeres**
  Leukocytes from FA patients can present relatively short telomeres, and FA cells are also characterised by telomere loss and/or break and increased level end-to-end telomere fusions that highlight a still poorly defined role of the FANC pathway in telomere maintenance [98, 99]. Several mechanisms were proposed to explain the observed abnormalities in the structure and functionality of the telomeres in FA cells, including DNA break accumulation at telomere sequences, accelerated replicative shortening due to unscheduled NHEJ activity, and impaired responses to oxidative stress. Moreover, some FANC proteins have been identified at telomeres: FANCD2, which colocalises with TRF1, FANCJ, a helicase involved in resolving G4 DNA structure and, recently, FANCM, whose depletion leads to ALT-specific telomeric replication stress [99, 100]. In addition, under replicative pressure, FANCC promotes short telomere maintenance in the absence of telomerase and its deficiency accelerates telomere attrition in bone marrow cells, potentially participating in the FANCC’s patient bone marrow failure [101].
  Together, these previous observations support a direct role of the FANC pathway, or some of its components, in telomere integrity and functions, whose loss not only increases genomic and telomeric instability but also causes the activation of the senescence programme.
- **Cellular and metabolic changes**
  FA cell lines of complementation groups A, C, D2 and G present mitochondrial dysfunction characterised by increased intracellular ROS levels, decreased mitochondrial potential, ATP production and oxygen uptake and changes in mitochondrial morphology [102, 103]. These activities are associated with the inactivation of enzymes essential for energy production (as cytochrome C oxidase) and detoxification of ROS (superoxide dismutase) [102]. Several FANC proteins interact biochemically or functionally with enzymes involved in redox homeostasis that are altered in FA cells. FANCA, FANCC, and FANCG are associated with cytochrome P450-related activities and/or respond to oxidative damage. FANCD2 interacts with FOXO3 in response to oxidative stress [104, 105]. FANCG interacts with peroxiredoxin 3, and FANCJ is a repressor of heme oxygenase-1 gene and sense oxidative base damage [103]. FANCD2, which interacts with the mitochondrial membrane ATP synthase ATP5α, appears to be involved in mitochondrial energy metabolism and mitochondrial gene transcription and translation [106, 107]. Mutations in these FANC components lead to the deregulation of mitochondrial homeostasis associated with inflammation and subsequent increased intracellular ROS levels. Moreover, via the FANCC-Parkin interaction, the FANC pathway appears to be involved in mitochondria turn-over, and its loss-of-function leads to alterations in the process of mitophagy, which is responsible for the clearance of damaged mitochondria [108]. Recently, it has been demonstrated that FANCD2 modulates mitochondrial stress response to prevent common fragile site instability [87].
  Recent observations have indicated that at least FANCA and FANCI are involved in ribosome biogenesis and mRNA translation with still unappreciated consequences on the metabolism of FA cells and development of the clinical traits of the syndrome [109].
  Newly, it has been demonstrated that hematopoietic differentiation is associated to transient stem cells transcription reprogramming which leads to R-loops formation and nuclear formaldehyde overload. Thus, the spontaneously generated high level of formaldehyde results in DNA and/or proteins crosslink whose repair requires FANC pathway. Notably, it has been described that ADH5 loss-of-function, that leads to intracellular formaldehyde overload and associated increased DNA damage, is lethally synthetic in a Fancd2-KO background [110]. On the basis of the previous observations, it has been proposed that the progressive and general attrition of blood/bone marrow cellularity in FA patients is due to the deleterious impact of endogenous produced formaldhyde causing aborted hematopoietic differentiation, DNA-damage-associated cell death and/or senescence [111].
  The described metabolic alterations in mitochondrial, energetic and ribosomal physiology observed in FA are also strongly associated with the senescence process in a p53-dependent and -independent manner.
- **Secretory phenotype**
  FA cells are characterised by altered responses to and/or overexpression of several lymphokines, cytokines and growth factors, including IL-1α, IL-6, TNF-α, TGF-β and interferons [112, 113]. At least some previous factors are also involved in DDR anomalies and their neutralisation with specific antibodies or inhibitors significantly reduces both FA cell chromosome fragility and hypersensitivity to treatment with ICL-inducing drugs [113]. The origin of the cytokine and growth factor overexpression/responses remains debatable and could represent a “physiological” response to palliate bone marrow failure, induced downstream of the intracellular accumulation of ROS, or induced by the presence of cytosolic DNA, or due to the lack of a canonical role of some FANC protein (not DNA repair related) in transcription or translational control. The unscheduled activation of several intracellular stress signalling pathways, including NF-κB, ERK, Jun and p38-MAPK, in turn could contribute to cytokine and growth factors hypersecretion.
  Indeed, FA cells are characterised by an increased level of both DNA damages and intracellular ROS that both can contributes to stress signals activation leading to pro-inflammatory cytokines production [114]. For instance, overexpression can be due to NF-κB-dependent matrix metalloprotease MMP-7 overexpression and/or the loss-of-function of FANCD2 that suppresses TNF-α gene expression linking a consensus element in the TNF-α promoter [115]. TNF-α overexpression, in turn, amplifies stress signalling pathway activation, the ROS level and mitochondrial dysfunction [116].
  In FA cells, the presence of DNA in the cytosol, due to the alterations in DNA repair, can contribute to both IFN signalling overactivation and IFN secretion via the cGAS/STING pathway, a key cell defence against virus infections [117, 118].
  Finally, the activation of stress signalling pathways and the secretion of several cytokines or growth factors could be a futile and pernicious attempt to rescue hematopoietic impairment and/or pancytopenia that characterise FA patients.
  All the previous pro-inflammatory mediators and stress signals are produced and activated during the senescence programme and can induce the senescence process in target cells. Thus, their overproduction in FA could lead to senescence either directly or by modifying the DDR capabilities of the FA cells.

### Direct evidence

*MiTF* is a key transcription factor involved in melanocyte, mast cell and osteoclast biology. Its unscheduled overexpression sustains melanoma progression and invasiveness, whereas its siRNA-mediated depletion in melanoma leads to genetic instability, mitotic abnormalities, growth arrest and senescence associated with the downregulation of FANC genes, known be among its direct targets. Notably, several consequences of MiTF depletion in melanoma were recapitulated by FANCA or FANCD2 depletion despite the maintenance of MiTF expression; inversely, their overexpression limits the consequence of MiTF depletion on melanoma cell behaviour [92].

We have recently demonstrated that FANC pathway depleted cells shown senescence hallmarks and phenotypes. In response to the unscheduled expression of an oncogene, FANCA and FANCD2 were first activated (to counteract the replication stress caused by the activated oncogene) before being actively degraded to allow senescence progression. Interestingly, FANCA and FANCD2 downregulation precedes p53, p21 and p16 activation. FANCD2 ectopic overexpression delays OIS progression without a major effect on p53 or p21 induction [93]. In FANCD2-depleted cells, an anticipated activation of CTSL1 was observed in parallel with the accelerated rise in senescence initiation [93]. A similar outcome was also observed for BRCA1, another FANC pathway-associated protein (FANCS) [119]. CTSL1 activation may orchestrate the arrest of futile DNA repair activities, permanently stopping cell proliferation and pushing the cells into senescence.

## Conclusion: FA as a “senescence disease”

The main downstream role of proteins of the FANC pathway is to repair cross-linked DNA and rescue delayed/blocked replication forks while maintaining genomic stability, and the multiple clinical and cellular phenotypes that define FA may be caused by a single factor with multiple tissue, cellular, biochemical and molecular consequences: the unscheduled activation of the senescence programme as a major consequence of the DNA damage-induced ATM-p53-p21 axis.

Moreover, it is tempted to speculate that the pro-senescent phenotype of the FA cells becomes, paradoxically, a driving force to select rare pre-leukemic cells that can overcome the growth-inhibited status characteristic of FA cells: the “dark side” of senescence (Fig. 5). Rare senescent cells could escape senescence and re-enter S-phase of the cell cycle using different mechanisms, such as transient inactivation of ATM, or ATR, combined with CHK1 and CHK2 inactivation, mutation acquisition in key proteins associated with senescence or reactivation of telomerase expression [120]. However, in DNA repair/DDR proficient cells, the escape frequency from OIS is estimated to be 1 in 10^6^ cells [121], which is more probable in cells with a pre-existing DNA repair or DDR deficit.

The FA clinical phenotype is characterised by abnormal embryo development involved in birth defects that alters several organs, including the skeleton, kidney and heart. These developmental defects may be caused by the loss of the organism’s normal ability to sculpt itself owing to the senescence and programmed cell death processes that represent two alternatives for the same purpose and that are abnormally active in FA cells.

The constitutive expression of the SASP, with multiple helpful effects when optimally and transiently regulated in a physiological setting, becomes unsafe with amplification of hematopoietic stem cell attrition, a pro-inflammatory status, DNA repair impairment and, finally, sustained cancer cell growth and expansion. Our hypothesis also furnishes a possible explanation of why *Fanc-*deficient mice generally present a mild FA phenotype compared with FA patients, despite similar hypogonadism/fertility reduction [122], impairment in the DNA repair and DDR. Compared with human cells, the telomeres in mice are longer and, to attain their critical shortening, two or three generations or inactivating mutations are needed in telomeric proteins. We speculate that the absence of telomeric abnormalities in FA mice reduces the intensity of the signal that leads to senescence activation (for example, reduced or absent secretion of pro-inflammatory cytokines, such as TNF-α of TGF-β) reduce the penetrance of the clinical stigma in the affected animals.

Thus, considering the involvement of FANC proteins in the control of the senescence programme and potential consequences of the deregulation of the latter for the FA phenotype, we propose that FA should be considered not only a DNA repair and chromosome fragility syndrome but also a cellular senescence-associated illness.
